# The predictive value of conventional surgical risk scores for periprocedural mortality in percutaneous mitral valve repair

**DOI:** 10.1007/s12471-016-0841-7

**Published:** 2016-05-17

**Authors:** F. A. Kortlandt, C. C. van ’t Klooster, A. L. M. Bakker, M. J. Swaans, J. C. Kelder, T. L. de Kroon, B. J. W. M. Rensing, F. D. Eefting, J. A. S. van der Heyden, M. C. Post

**Affiliations:** Department of Cardiology, St. Antonius Hospital, Nieuwegein, The Netherlands; Department of Cardio-Thoracic Surgery, St. Antonius Hospital, Nieuwegein, The Netherlands

**Keywords:** Mitral valve repair, Percutaneous, EuroSCORE, STS

## Abstract

**Background:**

Surgical risk scores are used to identify high-risk patients for surgical mitral valve repair. There is no scoring system to estimate the mortality risk for patients undergoing percutaneous treatment. The aim of this analysis is to evaluate the predictive value of the EuroSCOREs and the Society of Thoracic Surgeons Predicted Risk of Mortality Score (STS) for periprocedural mortality in percutaneous edge-to-edge mitral valve repair.

**Methods:**

From 2009 to 2013, 136 high-risk patients were included who underwent 143 procedures. Observed periprocedural mortality was compared with predicted mortality using the logistic EuroSCORE, EuroSCORE II and STS. The predictive value was analysed by receiver operating characteristic curves for each score.

**Results:**

Observed periprocedural mortality was 3.5 %. The predicted surgical mortality risk was: 23.1 ± 15.7 % for the logistic EuroSCORE, 9.6 ± 7.7 % for the EuroSCORE II and 13.2 ± 8.2 % for the STS. The predictive value estimated by the area under the curve was: 0.55, 0.54 and 0.65 for the logistic EuroSCORE, EuroSCORE II and STS respectively. Severe pulmonary hypertension and acute procedural success were significant predictive parameters in univariate analysis.

**Conclusion:**

Contemporary surgical scores do not adequately predict periprocedural mortality for high-risk patients undergoing edge-to-edge mitral valve repair, but they can be used to help decision-making in the selection process for this procedure.

## Background

Mitral regurgitation (MR) is the second most prevalent valve disease after aortic stenosis [1]. If untreated, severe MR can lead to progressive heart failure and reduced survival [2]. Currently, the standard for MR treatment is surgical mitral valve (MV) repair [3]. However, for many patients with severe MR, the surgical risk is considered too high. In these patients, percutaneous treatment of MR is an alternative therapy [4]. The best-established technique to date is the transcatheter edge-to-edge MV repair with the MitraClip® system (Abbott Vascular Inc. Santa Clara, CA, USA), which creates a double orifice, mimicking the surgical edge-to-edge technique introduced by Ottavio Alfieri [5]. The first in-human implantation was performed in June 2003 [6]. Since then, several studies have proven the safety and efficacy of this percutaneous treatment [7–14]. High-risk surgical or inoperable patients are potential candidates for this procedure. In current practice, the technique is predominantly used in the presence of functional MR [13].

To identify high-risk or inoperable patients who might be eligible for percutaneous MV repair, different well-established surgical risk models can be used to estimate operative mortality. The EuroSCOREs and the Society of Thoracic Surgeons Predicted Risk of Mortality Score (STS) are mostly used [4]. The logistic EuroSCORE was published in 1999 and is based on an international European database including patients predominantly undergoing coronary artery bypass graft surgery [15, 16]. A revised version, the EuroSCORE II, was developed in 2011 [17]. For surgically treated patients, Noyez et al. have proven the latter to be a more accurate version with a reduction of predicted risk of about 50 % [18]. The STS dates from January 2008 and includes a model specifically applicable for MV repair [19]. All of the three scoring models define perioperative mortality as in-hospital or 30-day mortality. As these models aim to predict mortality in cardiac surgery, they may not be applicable for percutaneous MV repair. Despite the fact that the percutaneous procedure is considered to be a safer alternative for mitral surgery, it can still be accountable for potentially life-threatening complications [20]. Reliable estimation of the mortality risk would contribute to adequate patient selection for this procedure. The aim of this retrospective observational study is to examine the predictive value of the EuroSCOREs and the STS for periprocedural mortality in percutaneous MV repair and to identify other predictors.

## Methods

### Study population

From January 2009 to April 2013, 143 percutaneous MV procedures were performed in 136 consecutive patients at our institution. The Heart Team referred patients for percutaneous MV repair after careful deliberation. All patients suffered from moderate-to-severe (3+) or severe (4) MR, according to the recommendations of the American Society of Echocardiography [21], and were considered high risk for conventional surgery (logistic EuroSCORE > 20 % or the presence of specific risk factors for morbidity or mortality decided by the Heart Team). Baseline characteristics, including medical history, laboratory findings and New York Heart Association (NYHA) classification, were collected and entered into a web-based electronic registry. The local institutional review board approved the study protocol (R&D/Z-13.15/MitraClip).

### Risk scores

The logistic EuroSCORE, EuroSCORE II and STS were calculated using online calculators (http://riskcalc.sts.org and http://www.euroscore.org, respectively). The two EuroSCOREs are subdivided into three categories (patient related, cardiac related and operation related), and comprise 17 and 18 variables for the logistic EuroSCORE and EuroSCORE II, respectively. The STS is divided into 8 categories (procedure, demographics, risk factors, previous interventions, preoperative cardiac status, preoperative medications, haemodynamics and catheterisation, and valve pathology) and consists of 41 variables. In the STS, MV repair was specifically indicated. Frequently used cut-off values to define high surgical risk for the logistic EuroSCORE (≥20) and the STS (≥12) were applied [12, 13]. For the EuroSCORE II no commonly used limit is available, therefore data were arbitrarily analysed for the threshold values of ≥5 and ≥10 %.

### Procedural technique

Percutaneous MV repair was performed by means of the MitraClip® system, as previously described [9].

### Endpoints definition

The main endpoint of this analysis was periprocedural mortality, defined as 30-day mortality or in-hospital death after the procedure, similar to the definition used in the risk scores.

### Statistical analysis

Descriptive statistics were used to report patient characteristics. Continuous variables were reported as mean ± standard deviation. Frequencies and percentages were used to report nominal variables. The prognostic value of the EuroSCOREs and the STS was evaluated in terms of discrimination, assessed by a receiver operating characteristic (ROC) analysis, producing an area under the curve (AUC) with probability values (*p*-value). A two-sided *p*-value of < 0.05 was considered significant. Furthermore, the mortality ratio was calculated by dividing the observed mortality by the predicted mortality multiplied by 100. This was done for the different cut-off values. If this ratio equals 100 %, then the number of observed deaths equals that of predicted cases. To identify other risk factors, patient characteristics of the periprocedural mortality group were compared with survivors using the Fisher’s exact test for nominal variables and the independent Student’s t‑test for continuous variables. All statistical analyses were performed using SPSS software (SPSS version 21.0 for Windows, IBM, Armonk, New York) and R (version 2.15, www.r-project.org).

## Results

A total of 143 percutaneous MV procedures were performed in 136 patients (mean age: 74.5 ± 9.4 years, male: 67.6 %, Tab. 1). Acute procedural success, defined as a reduction of the MR to grade ≤ 2+, was achieved in 132 of 143 procedures (92.3 %) [14]. Due to a failed index procedure or recurrent MR, 7 patients underwent a “redo” procedure. Furthermore, in 5 patients no clip was implanted for different reasons: either no reduction of the MR could be achieved after grasping the leaflets (*n* = 2) or the patient’s anatomy proved unsuitable for MitraClip ® treatment (*n* = 2). One patient died before clip placement during an urgent redo procedure due to worsening MR after myocardial infarction. All procedures, including failed procedures and redo procedures, were reported and analysed, thus examining the periprocedural mortality for every procedure. Within 30 days, 5 deaths had occurred. None of the patients who were alive at 30 days died before hospital discharge, resulting in a periprocedural mortality rate of 3.5 %. The cause of death was cardiovascular in all cases; 4 patients died due to end-stage heart failure and 1 patient died due to a sudden cardiac arrest without preceding symptoms of heart failure.

**Tab. 1:** Pre-procedural patient characteristics

	*n* = 136
**Patient-related factors**	
Age, years	74.5 ± 9.4
Male	92 (67.6)
BMI, kg/m^2^	25.9 ± 4.7
**Comorbidities**	
Diabetes mellitus	31 (22.8)
Hypertension	70 (51.5)
Atrial fibrillation	72 (52.9)
COPD	28 (20.6)
Renal failure^a^	54 (39.7)
Previous myocardial infarction	69 (50.7)
Previous CABG	58 (42.6)
Previous MV repair	2 (1.5)
Stroke/TIA	20 (14.7)
**Performance**	
NYHA III or IV	122 (89.7)
Quality of life index score	50 ± 23
6-MWT distance, m	273 ± 120
**Laboratory findings**	
NT proBNP, pg/ml	4591 ± 6178
Haemoglobin, mmol/l	7.9 ± 1.0
Creatinine, µmol/l	132.5 ± 68.1
***Cardiac-related factors***	
*MR aetiology*	
Degenerative	23 (16.9)
Functional	106 (77.9)
Mixed	7 (5.1)
LVEF, %	36.4 ± 15.3
*Pulmonary hypertension* ^b^	
Moderate	96 (70.6)
Severe	25 (18.4)
Resynchronisation therapy	30 (22.1)

Values are mean ± SD or *n* (%)

*BMI* body mass index, *CABG* coronary artery bypass graft, *COPD* chronic obstructive pulmonary disease, *CRT* cardiac resynchronisation therapy, *LVEF* left ventricle ejection fraction, *MR* mitral regurgitation, *NYHA* New York Heart Association, *TIA* transient ischaemic attack, *6-MWT* 6-minute walk test

^a^Glomerular filtration rate < 45 ml/min/1.73 m^2^

^b^According to the two classes in the EuroSCORE II: moderate pulmonary hypertension is a systolic pulmonary artery pressure (sPAP) between 31–55 mmHg, severe pulmonary hypertension is a sPAP above 55 mmHg

### EuroSCOREs and STS score analysis

The overall 30-day or in-hospital risk of mortality estimated by the logistic EuroSCORE, EuroSCORE II and STS was 23.1 ± 15.7 %, 9.6 ± 7.7 % and 13.2 ± 8.2 %, respectively (Tab. 2). Since the observed mortality was 3.5 %, all scores overestimated the risk of mortality.

**Tab. 2:** The prognostic value of the logistic EuroSCORE, EuroSCORE II and the STS score

	Predicted mortality	AUC (95 % CI)	*p*-value
LES	23.1 ± 15.7	0.55 (0.32–0.78)	0.70
ES II	9.6 ± 7.7	0.54 (0.30–0.77)	0.78
STS	13.2 ± 8.2	0.65 (0.49–0.81)	0.25

Values are mean ± SD

*LES* Logistic EuroSCORE, *ESII* EuroSCORE II, *STS* Society of Thoracic Surgeons Predicted Risk of Mortality Score, *AUC* area under the curve, *CI* confidence interval

**Fig. 1:**
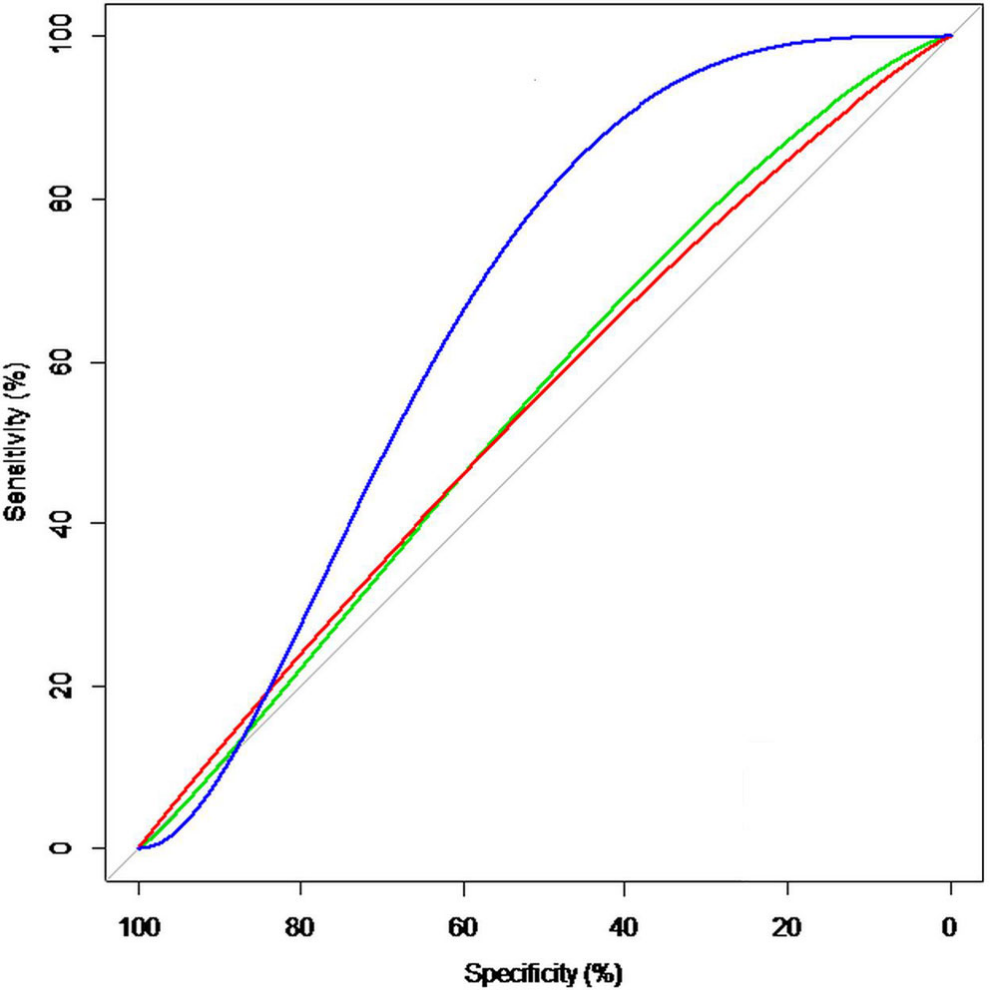
ROC curve analysis for the prediction of periprocedural mortality. *Green line* LES: 55.1 %, *red line* ES II: 53.6 %, *blue line* STS: 65.1 %

The discriminating value of the three scores was examined in an ROC curve analysis for the prediction of periprocedural mortality (area under the curve: 0.55 (*p* = 0.69) for the logistic EuroSCORE vs 0.54 (*p* = 0.78) for the EuroSCORE II vs 0.65 (*p* = 0.25) for the STS, Tab. 2 and Fig. 1). Although the STS outperformed both EuroSCORES, none of the scores showed a significant correlation with periprocedural mortality.

When applying different cut-off values for the identification of very high-risk patients, the association between the observed and predicted mortality was poor. The EuroSCORE II with a cut-off value of ≥5 % showed the highest mortality ratio of 32.6 (Tab. 3).

**Tab. 3:** The observed to predicted mortality for different cut-off values for peri-procedural mortality

Cut-off value	% of procedures	Observed	Mortality Predicted	Ratio
LES ≥ 20	49.6	4.2 (0.9–11.9)	35.1	12.0
ES II ≥ 5	67.1	4.2 (1.1–10.3)	12.9	32.6
ES II ≥ 10	39.7	3.7 (0.5–12.7)	17.5	21.1
STS ≥ 12	47.5	5.9 (1.6–14.2)	20.0	29.5

Values are % (95 % confidence interval)

*LES* Logistic EuroSCORE, *ESII* EuroSCORE II, *STS* Society of Thoracic Surgeons Predicted Risk of Mortality Score

The predictive value of each baseline characteristic was assessed in univariate analysis. Severe pulmonary hypertension was identified as a predictor for perioperative mortality (OR 7.5; CI 95 % 1.2–47.4, *p* = 0.04). Furthermore, patients with acute procedural success showed significantly lower perioperative mortality (OR 0.1; 95 % CI 0.02–0.7, *p* = 0.05). NYHA functional class also seemed to predict periprocedural mortality due to statistical significance, but it has less predictive value due to an odds ratio near 1 (OR 1.08, 95 % CI 1.00–1.16, *p* = 0.04).

## Discussion

The aim of this observational study was to examine the predictive value for periprocedural mortality of the conventional surgical risk scores in high-risk patients undergoing percutaneous MV repair.

The observed periprocedural mortality was 3.5 % in 143 procedures. Our results show a distinct overestimation of the mortality risk by all three risk models. Also, when cut-off values were applied, the mortality ratio was nowhere near 100 %, which confirms that the scores are not accurate in predicting periprocedural mortality in percutaneous MV repair. These findings are unsurprising, since the surgical risk scores were designed to predict mortality in patients undergoing open-heart surgery instead of percutaneous procedures. Hence, it is not only expected, but also required that the scores overestimate the actual periprocedural mortality risk. However, an earlier analysis by our group showed that patients with high-surgical risk (mean logistic EuroSCORE of 33.8 ± 9.0 %) who are denied surgical treatment can be successfully treated by using percutaneous MV repair leading to a 30-day mortality of 0 % [22]. The same is true for a study by Pleger et al. [8], with a mean logistic EuroSCORE of 41 ± 7 % and 30-day mortality of 0 %. Thus, the risk scores can be useful in identifying high-risk surgical patients in order to select them for percutaneous MV treatment as a safer alternative for surgery.

Furthermore, the two EuroSCOREs and the STS score date from 1999, 2011 and 2008 respectively [15–17, 19]. With surgical techniques and postoperative care improving, the scores are apt to overestimate the risk of perioperative mortality [23].

Previous studies show similar results: the large ACCESS-EU registry showed a periprocedural mortality rate of 3.4 % with a mean logistic EuroSCORE of 23.0 ± 18.3 % and the TRAMI registry showed a periprocedural mortality of 2.5 % with a median logistic EuroSCORE of 23.0 % (interquartile range of 12–38 %) and a median STS of 11.0 % (interquartile range of 4–19 %) [11, 13]. Recently, a publication from the GRASP-IT registry also stated that all three scores overestimated mortality at 30 days with an observed mortality rate of 3.3 % (mean of 17 ± 5 %, 7 ± 8 % and 8 ± 7 % for logistic EuroSCORE, EuroSCORE II and STS respectively). All scores could distinguish between low- and high-risk groups at 3‑year follow-up, the EuroSCORE II and logistic EuroSCORE also being able to discriminate at a 30-day term. However, none of the scores were correctly calibrated for periprocedural mortality rate [24].

These studies, in agreement with our analysis, prove that the surgical risk scores overestimate the 30-day observed mortality rate in percutaneous MV repair.

Even though percutaneous MV repair proves to be relatively safe, life-threatening complications can occur [20]. A dedicated risk model for the percutaneous MV procedure might facilitate patient selection. In this observational study, only severe pulmonary hypertension could be identified as a significant and relevant pre-procedural predictor for periprocedural mortality in univariate analysis. This outcome made development of a mortality prediction tool for percutaneous MV repair unattainable and beyond the scope of this analysis. Several other studies have identified variables to be predictors for mortality such as: elevated NT-proBNP, chronic obstructive pulmonary disease, kidney failure, previous valve surgery, tricuspid valve insufficiency, NYHA IV and pre-procedural MR grade [25–29]. However, so far there remains little consistency and studies have been conducted with too few patients to mimic the realisation of a model such as the EuroSCORE [15, 30]. A well-designed study with a larger dataset and more events could be able to develop such a risk score for percutaneous MV repair.

## Limitations

The data were gathered from a single centre, which might induce selection bias by the heart team process. Additionally, since the observed periprocedural mortality was low (3.5 %), a larger sample size could have led to more reliable results.

## Conclusion

The results of this analysis show that conventional risk scores overestimate the periprocedural mortality in patients undergoing percutaneous MV repair. However, the risk scores are useful in identifying eligible patients for percutaneous treatment. A dedicated risk model for estimation of the mortality risk for percutaneous MV repair could optimise patient selection for this procedure.
